# Novel chimeric antigen receptor T cell-based immunotherapy: a perspective for triple-negative breast cancer

**DOI:** 10.3389/fcell.2023.1158539

**Published:** 2023-06-29

**Authors:** Peizhen Geng, Yuhua Chi, Yuan Yuan, Maoquan Yang, Xiaohua Zhao, Zhengchun Liu, Guangwei Liu, Yihui Liu, Liang Zhu, Shuai Wang

**Affiliations:** ^1^ School of Clinical Medicine, Affiliated Hospital of Weifang Medical University, Weifang Medical University, Weifang, Shandong, China; ^2^ Department of General Medicine, Affiliated Hospital of Weifang Medical University, Weifang, Shandong, China; ^3^ Department of Thoracic Surgery, Affiliated Hospital of Weifang Medical University, Weifang, Shandong, China; ^4^ Key Laboratory of Precision Radiation Therapy for Tumors in Weifang City, Department of Radiotherapy, School of Medical Imaging, Affiliated Hospital of Weifang Medical University, Weifang Medical University, Weifang, Shandong, China; ^5^ Clinical Research Center, Department of Endocrinology and Metabolism, Affiliated Hospital of Weifang Medical University, Weifang, Shandong, China

**Keywords:** triple negative breast cancer, tumor antigens, immunotherapy, chimeric antigen receptors, radiotherapy

## Abstract

Triple-negative breast cancer (TNBC) is highly aggressive and does not express estrogen receptor (ER), progesterone (PR), or human epidermal growth factor receptor 2 (HER2). It has a poor prognosis, and traditional endocrine and anti-HER2 targeted therapies have low efficacy against it. In contrast, surgery, radiotherapy, and/or systemic chemotherapy are relatively effective at controlling TNBC. The resistance of TNBC to currently available clinical therapies has had a significantly negative impact on its treatment outcomes. Hence, new therapeutic options are urgently required. Chimeric antigen receptor T cell (CAR-T) therapy is a type of immunotherapy that integrates the antigen specificity of antibodies and the tumor-killing effect of T cells. CAR-T therapy has demonstrated excellent clinical efficacy against hematological cancers. However, its efficacy against solid tumors such as TNBC is inadequate. The present review aimed to investigate various aspects of CAR-T administration as TNBC therapy. We summarized the potential therapeutic targets of CAR-T that were identified in preclinical studies and clinical trials on TNBC. We addressed the limitations of using CAR-T in the treatment of TNBC in particular and solid tumors in general and explored key strategies to overcome these impediments. Finally, we comprehensively examined the advancement of CAR-T immunotherapy as well as countermeasures that could improve its efficacy as a TNBC treatment and the prognosis of patients with this type of cancer.

## 1 Introduction

Global Cancer Statistics 2020 reported 2.261 million new cases of breast cancer (BC) worldwide. The overall incidence of BC was significantly higher in developed than developing countries (Sung et al., 2021). BCs are now some of the most frequently diagnosed malignant tumors, and it accounts for a substantial proportion of all cancer-related deaths in human females. Improvements in early diagnosis, surgical techniques, chemotherapy, and radiotherapy have considerably lowered the mortality rate of BCs. Nevertheless, its prognosis remains poor (Everatt and Gudaviciene, 2022). In this disease, normal breast cells are transformed into cancer cells that clone, proliferate, invade, and metastasize without any external stimuli (Evan and Vousden, 2001). Aberrant alteration of normal transformed myoepithelial or epithelial cells and/or transformation of stem cells with low differentiation capacity may explain the characteristic heterogeneity of BCs (Gusterson et al., 1982).

BCs express estrogen receptor (ER), progesterone receptor (PR), and human epidermal growth factor receptor 2 (HER2) at different levels, and their proliferation indices (such as Ki-67) vary as well. Hence, BCs have been classified into four primary molecular subtypes, namely, Luminal A (ER+, PR+, HER2-, Ki-67 < 14%), Luminal B (ER+, PR+, HER2+ or HER2-, Ki-67 > 14%), HER-2 overexpression (ER-, PR-, HER2+), and basal-like (ER-, PR-, HER2-) (Network, 2012). All subtypes except basal-like BC responded favorably to treatment with paclitaxel, anthracyclines, and alkylating agents as well as HER2-targeting monoclonal antibodies and endocrine therapy (Kennecke et al., 2010; Kim et al., 2020). Triple-negative breast cancer (TNBC), accounting for approximately 15%–20% of all BC cases, is defined as lacking the expression of ER, PR, and HER2 (Schmadeka et al., 2014). There is an 80% match between TNBC and basal-like BC, and clinicians often use the triple negative definition as a surrogate for basal-like breast cancer (Goldhirsch et al., 2013). Due to the malignant progression, the high tumor grade, and the absence of specific targets of TNBC, the US Food and Drug Administration (FDA) has not developed a targeted therapy for TNBC. The prognosis for BC patients who have undergone surgery and cytotoxic chemotherapy is poor. Metastatic recurrence, few treatment options, low response rates, and lack of persistence result in short survival (Kumar and Aggarwal, 2016; Jia et al., 2017). Therefore, novel treatment options that significantly improve outcomes are urgently needed for TNBC. We will now describe the implementation of chimeric antigen receptor (CAR) technology as an immunocyte therapy targeting TNBC. We will address potential therapeutic targets of CAR-T in TNBC, evaluate this approach in preclinical studies and clinical trials, examine the barriers and obstacles associated with the administration of CAR-T against TNBC and other solid tumors, and review key strategies that may overcome these limitations.

## 2 CAR-T cell immunotherapy and CARs

**FIGURE 1:**
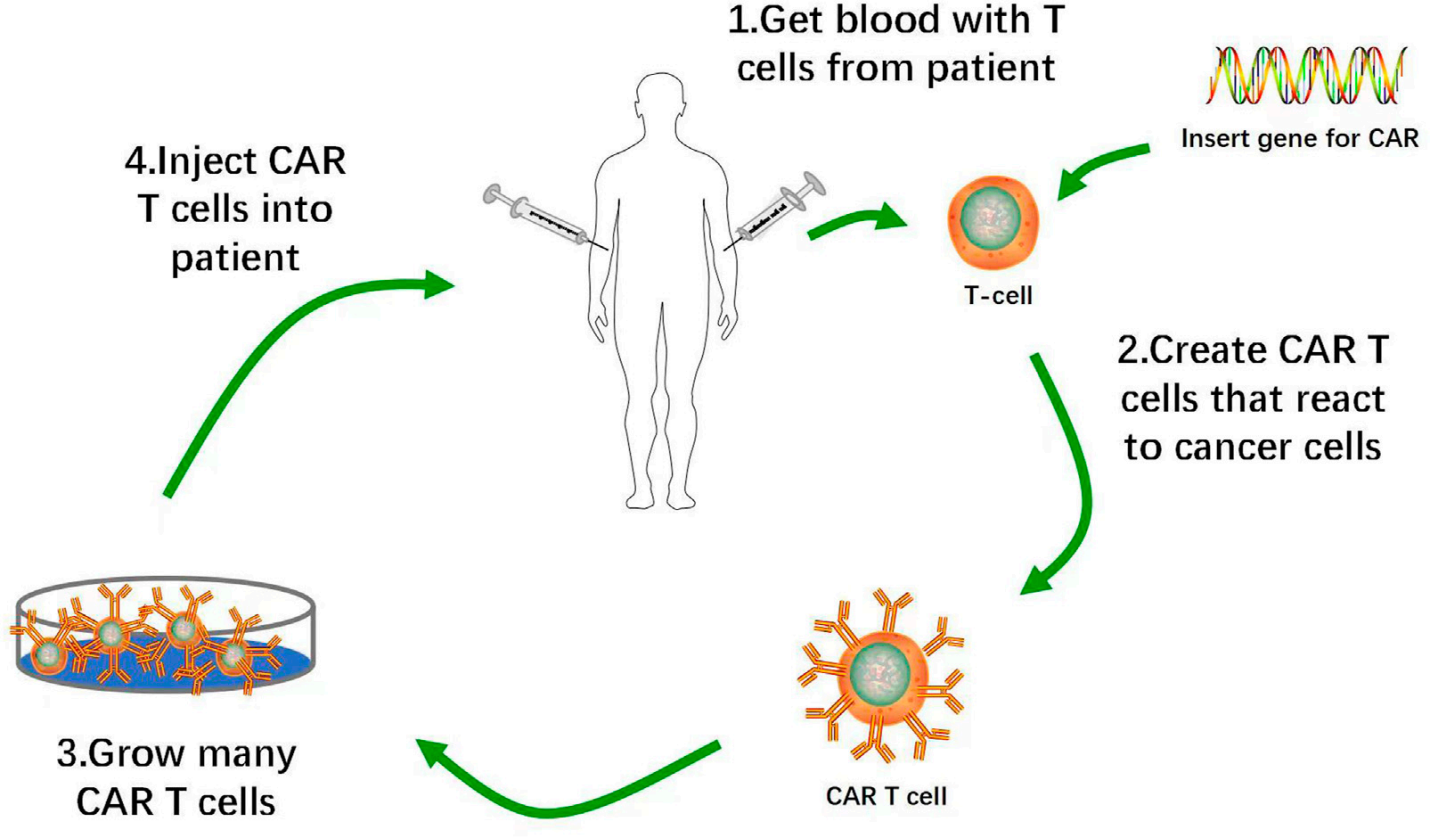
Flow of Production of CAR-T Cells. 1. Removal of T cells from patient with cancer. 2. Activation of T cells with anti-CD3 anti-CD28 activation beads, and transduction of activated T cells with CAR construct. 3. *Ex vivo* expansion of CAR-T cells and removal of activation beads. 4. Administration of lymphodepletion chemotherapy followed by infusion of CAR-T cell product into patients.

CAR-T cells constitute a promising immunotherapy that has effectively treated hematological malignancies in clinical trials. CAR-T cell therapy targeting Cluster of Differentiation (CD)19 inhibits the progression of B cell malignancies and has significantly improved the prognosis of many patients with relapsed or refractory B cell acute lymphoblastic leukemia (B-ALL). CAR-T cell therapy induced morphologically complete remission in 70%–90% of B-ALL patients (Maude et al., 2014; Lee et al., 2015). T lymphocytes isolated from autologous peripheral blood were genetically engineered to express CARs on their membranes, amplified *in vitro*, and transfused back into the patients. CARs mediate tumor surface-associated antigen recognition in the major histocompatibility complex (MHC)-unrestricted manner, activate T lymphocytes, and cause tumor killing (Figure 1) (Eshhar et al., 1993; Feins et al., 2019).

CARs are artificial fusion proteins that usually include extracellular antigen recognition structural domains derived from single-chain variable fragments (scFv) of antibodies (Kochenderfer and Rosenberg, 2013), spacer/hinge structural domains that regulate CAR flexibility and optimize cell-to-cell distances, transmembrane structural domains that anchor CARs to T cell membrane surfaces, and intracellular signaling structural domain co-stimulatory molecules (Bridgeman et al., 2010; Hombach et al., 2010). Single-variable domains on a heavy chain (VHH; nanobodies) are derived from the CAR targeting domains. The results of preclinical and clinical studies showed that VHH-based CAR-Ts may mediate specific target antigen-dependent cytotoxicity against various malignant tumors. Compared with traditional full-length scFvs, nanobodies recognize and bind target antigens with similar binding abilities and specificities (Safarzadeh Kozani et al., 2022). Another study demonstrated that oligoclonal CAR-Ts with oligoclonal anti-HER2 nanobodies had comparatively elevated proliferation, cytokine secretion, and tumoricidal capacity. VHH-based CD19-redirected CAR-Ts can mediate targeted antitumor activity and efficient cytokine secretion, thereby achieving high tolerance and satisfactory complete remission (CR) rates (Nasiri et al., 2023).

**FIGURE 2:**
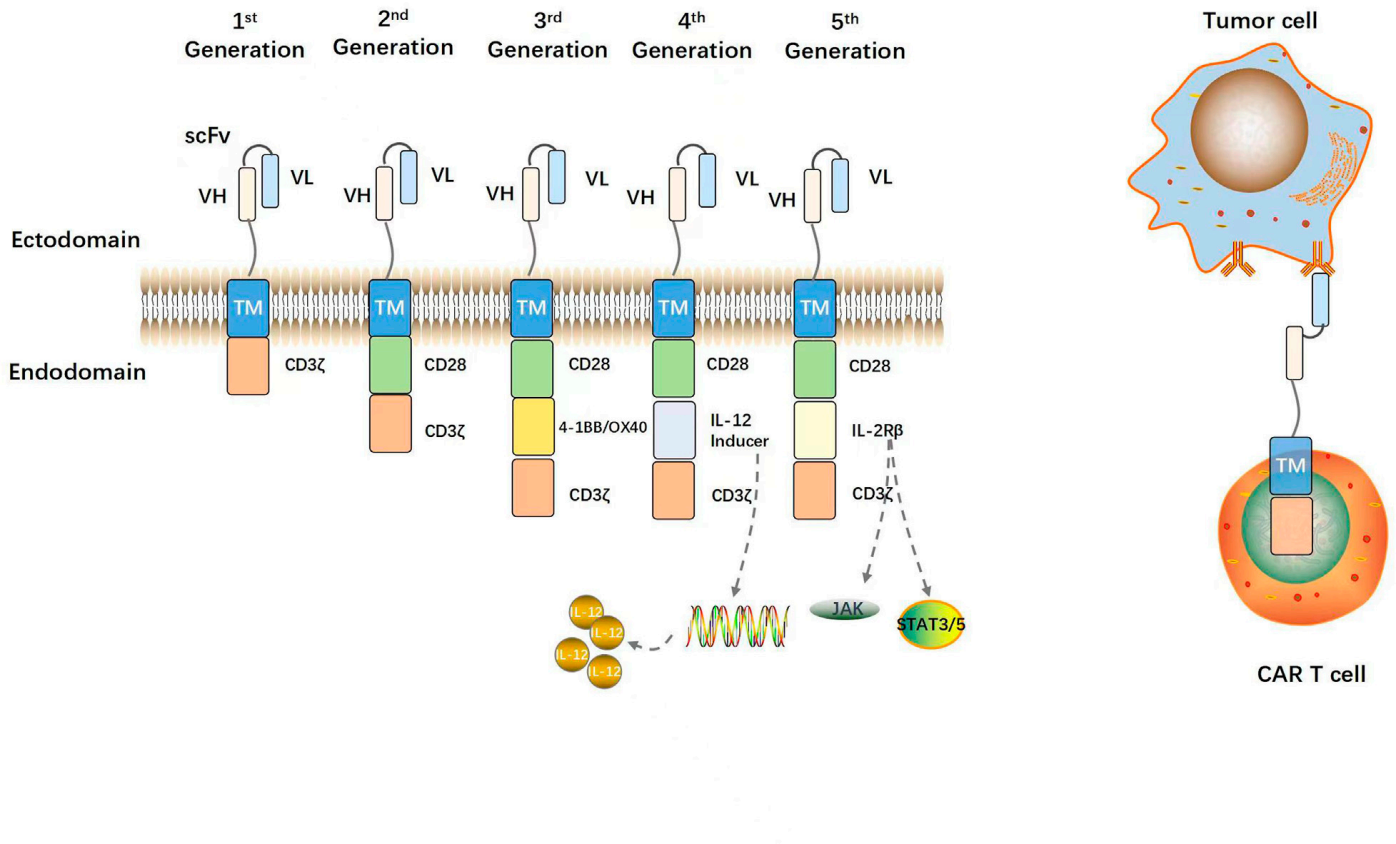
Structural evolution of CARs. A prototypical CAR consists of an extracellular antigen binding scFv (VH and VL regions of antibody linked by a glycine-serine peptide sequence), a flexible spacer or hinge region, a transmembrane domain, and an intracellular CD3ζ activation domain. First-generation CARs contain only a CD3ζ activation domain. Second-generation CARs contain one costimulatory domain (e.g., CD28). Third-generation CARs contain two costimulatory domains (e.g., CD28 and 4-1BB). The fourth- and fifth-generation CARs contain an interleukin expression inducer domain and an interleukin intracellular receptor, respectively. CAR-modified T cells are activated and can efficiently kill tumor cells via binding of the CAR to TAAs on tumor cells independent of MHC.

Figure 2 shows the evolution of CARs over several generations. Eshhar et al. (1993) constructed a chimeric molecule consisting of ScFv bound to the structural domain of the constant region in the T cell receptor (TCR). In this way, specificity of antibody and cytotoxicity of T cells and other effector lymphocytes, such as natural killer cells were realized. The ζ-chain TCR/CD3 complex of the antibody molecule might serve as a “first-generation CAR” (Eshhar et al., 1993). Though the CARs could repeatedly recognize antigens and activate T lymphocytes, they could not maintain long-term adaptive immune function and exhibit only weak antitumor ability (Kershaw et al., 2006). Co-stimulatory signaling domains such as CD28, CD27, or 4-1BB (CD137) were then added to the “first-generation CARs” to create “second-generation CARs”. The structural domain constitutively activates T cells, induces cytokine secretion, promotes T cell proliferation and anti-apoptosis, and improves antigen recognition and antitumor ability (Maher et al., 2002; van der Stegen et al., 2015). “Third-generation CARs” consist of at least two co-stimulatory domains that collaborate with a signaling domain (CD3ζ) and activate T-lymphocytes, and a co-stimulatory domain, usually CD28 and 4-1BB or OX40 (CD134) that augments T-lymphocyte function. Successive CAR-T cell generations might exhibit superior expansion and persistence in individual patients (Till et al., 2012; Cheng et al., 2018). Chmielewski et al. (2015) attempted to circumvent the substantial phenotypic heterogeneity characteristic of solid tumors by developing “fourth-generation CARs” or TRUCKs (T cells redirected against universal cytokine-mediated killing). The authors added a CAR-inducible interleukin (IL)-12 cassette to first/second-generation CARs (Chmielewski and Abken, 2015). When a CAR binds a tumor antigen, it activates downstream signaling pathways and promotes the secretion of the pro-inflammatory factors IL-12, IL-18, CD40L, IL-15, and IL-21 (Knochelmann et al., 2018). These interleukins then accumulate in the target area and recruit NK cells and macrophages. These innate immune cells kill target tumors that evade T cell attack and lack CAR recognition. They also mediate innate immune cell responses to cancer cells that are otherwise invisible to CAR-T cells (Chmielewski et al., 2014). “Fifth-generation CARs” are created by adding an IL-2 receptor β (IL-2Rβ) fragment to second-generation CARs. Fifth-generation CAR technology involves the Janus kinase (JAK) and the signal transducer and activator of transcription (STAT)3/5 binding sites. After CAR-T cells target tumor antigens, the JAK and STAT3/5 signaling pathways are immediately triggered by IL-2R β fragments, thereby augmenting the antitumor efficacy and *in vivo* persistence of T cells (Kagoya et al., 2018; Tokarew et al., 2019).

## 3 Potential preclinical CAR-T targets in TNBC

### 3.1 Folate receptor alpha (FRα)

FRα is a glycosyl-phosphatidyl inositol (GPI)-anchored glycoprotein in cell membranes. It is overexpressed in high-grade TNBC and accounts for 71%–86% of all metastatic TNBC (Cheung et al., 2018; Xie et al., 2020). FRα promotes TNBC cell division and migration by binding folate receptors and regulating the JAK/STAT and other cancer-related signaling pathways. In contrast, FRα inhibition has an important antitumor effect (Scaranti et al., 2020). FRα-CAR-T cells targeted FRα+ TNBC cells, killed TNBC cells *in vitro*, and caused significant tumor regression in a mouse MDA-MB-231 tumor xenograft model (Song et al., 2016). A randomized Phase II study on patients with TNBC demonstrated that the FRα polypeptide vaccine effectively prevented recurrence after Stage 1–3 TNBC surgery and other standard treatments (NCT03012100). A trans-signaling CAR strategy was adopted to mitigate FRα-related on-target/off-tumor toxicity. The CD3ζ and CD28 signaling domains were established in two separate CAR and T lymphocyte lines and they simultaneously targeted tumor cells containing the antigens mesothelin and FRα (Lanitis et al., 2013). The bispecific small molecule ligand EC17 and fluorescein isothiocyanate (FITC) bind folic acid, cause FITC-CAR-T cells to target tumor cells overexpressing FRα, prevent T cell depletion, and improve antigen control flexibility and safety (Lu et al., 2019). A fourth-generation FRα-CAR-T cell clinical trial in a Phase I/II multicenter study (NCT03185468) was conducted to evaluate locally advanced or metastatic uroepithelial bladder cancer (UBC). Though neither efficacy nor safety data have yet been reported, the favorable outcome of this trial might pave the way for the routine use of this approach in TNBC treatment.

### 3.2 Tumor endothelial marker 8 (TEM8)

TEM8 is a highly conserved integrin-like glycoprotein that is preferentially expressed in regions of aberrant neovascularization within a tumor. Targeted TEM8 silencing via the extracellular signal-related kinase (ERK)/B cell lymphoma 2 (Bcl-2) signaling pathway significantly inhibited tumor growth and was anti-angiogenic (Gong et al., 2021). TEM8 is highly expressed on TNBC cells and is a putative stem cell-like cell (BCSC) marker. As TEM8 is expressed on TNBC tumor cells, it might be suitable as an antigen for targeted immunotherapy. Second- and third-generation TEM8-specific CAR-T cells targeting TEM8 were cytotoxic to Hs578T, MDA-MB-231, MDA-MB-436, and MDA-MB-468 TNBC cell lines as well as the HC6020 human BC endothelial cell line. They also caused tumor regression in established TNBC xenografts (Byrd et al., 2018). In contrast, another study showed that mice treated with TEM8-specific CAR exhibited normal tissues with off-target toxicity, inflammation, and dense infiltrative neutrophil aggregates in the lungs and spleen (Petrovic et al., 2019). Moreover, mice administered with TEM8-specific human antibody (L2 fragment), which could activate secretory bispecific T lymphocytes, exhibited toxicity attributed to off-tumor/on-target TEM8. No toxicity was observed in TEM8 knockout mice (Petrovic et al., 2019). Therefore, the foregoing complications must be carefully considered when CAR-T therapies targeting TEM8 are implemented in clinical trials.

### 3.3 Chondroitin sulfate proteoglycan 4 (CSPG4)

CSPG4 is a highly glycosylated transmembrane protein consisting of an *N*′-linked, 280-kDa glycoprotein and a 450-kDa chondroitin sulfate proteoglycan (Campoli et al., 2004). As CSPG4 is an attractive target antigen in TNBC immunotherapy, its upregulation is correlated with an aggressive phenotype and poor prognosis. CSPG4-CAR-T cells concomitantly targeted CSPG4+ TNBC cells and cancer-associated fibroblasts (CAFs), and CSPG4 was detected in TNBC stem cells. Thus, CSPG4 might participate in cancer relapse and resistance and maintain the tumor microenvironment (TME) (Wang et al., 2010; Cooney et al., 2011; Hsu et al., 2013). CSPG4-specific monoclonal antibody (mAb) inhibited TNBC cell migration and growth, caused significant regression of lung metastasis, and limited tumor recurrence in a mouse immunodeficient *in situ* xenograft model. The CSPG4-specific mAb blocked the focal adhesion kinase (FAK), ERK1/2, protein kinase C alpha (PKCα), and protein kinase (Akt) signaling pathways regulating cell proliferation, adhesion, and migration (Wang et al., 2010). CSPG4 may induce on-target/off-tumor toxicity in TNBC immunotherapy and promote CSPG4-CAR in the pericytes around tumor-associated endothelial cells. As TNBC is genetically unstable, its treatment is challenged by antigen loss and non-uniform expression (Wang et al., 2010; Harrer et al., 2019).

### 3.4 Intercellular adhesion molecule-1 (ICAM-1)

The cell adhesion glycoprotein ICAM-1 promotes leukocyte and endothelial cell migration and stabilizes intercellular interactions (Hubbard AK and Rothlein, 2000). It stimulates cancer cell proliferation and migration and is anti-apoptotic as it activates multiple intracellular signaling pathways. ICAM-1 density and distribution on cell surfaces affect the transcellular transendothelial migration (TEM) (Yang et al., 2005). ICAM expression is higher in TNBC than in other BC subtypes or normal breast tissue (Hsu et al., 2013). Anti-ICAM-1 antibody significantly inhibited the invasion of the highly metastatic MDA-MB-435 cell line in a dose-dependent manner. Hence, ICAM-1 is a therapeutic target in TNBC (Rosette et al., 2005). ICAM1-specific CAR-T cells significantly reduced TNBC cell proliferation and extended long-term therapeutic efficacy and survival time in TNBC (Wei et al., 2020). A multicenter Phase I clinical trial (NCT04420754) has been launched to assess the safety and tolerability of autologous AIC100 CAR-T cells targeting ICAM-1 in undifferentiated, recurrent, and refractory advanced thyroid cancers (Ilieva et al., 2017). As ICAM-1-CAR-T has a significant killing effect on TNBC cells, it could be implemented in clinical trials.

### 3.5 Receptor tyrosine kinase (AXL/PTK)

AXL is a receptor tyrosine kinase that activates the downstream phosphoinositide 3-kinase (PI3K), mitogen-activated protein kinase (MAPK), and JAK/STAT signaling pathways. It is implicated in tumor cell survival, anti-apoptosis, migration, invasion, drug resistance, angiogenesis, and tumor-host relationship progression (Zhu et al., 2019). AXL was significantly upregulated in triple-negative/basal B cell line BCs compared to ductal and basal A cell line BCs (D'Alfonso et al., 2014). AXL was only weakly expressed in normal cells and tissues (Wei et al., 2018). AXL-CAR-T therapy significantly induced antigen-specific cytotoxicity and cytokine release in TNBC cell lines. It also displayed significant antitumor efficacy and caused T cell persistence in an MDA-MB-231 xenograft model (Wei et al., 2018). AXL-CAR-T inhibited tumor growth in the aforementioned model, enhanced the therapeutic efficacy of anti-vascular endothelial growth factor (VEGF) and anti-epidermal growth factor receptor (EGFR) small molecule inhibitors as well as chemotherapy in TNBC, and altered the tumor mesenchyme by modulating the TNBC-associated vascular system and immune cell function (Ye et al., 2010). As the AXL receptor is expressed in myeloid-derived suppressor cells (MDSCs), AXL-targeting CAR-T cells deplete the MDSC of TME and cause it to enter a pro-inflammatory state (Brenig et al., 2020). Combinations of AXL-CAR-T and constitutionally activated IL-7 receptor blockers exhibited strong antitumor activity and prolonged survival time *in vitro* (Zhao et al., 2020). Therefore, targeting AXL is a potential approach toward CAR-T cell therapy against TNBC.

### 3.6 Disialoganglioside (GD2)

The acidic glycosphingolipid GD2 facilitates interactions between tumor cells and extracellular matrix (ECM) proteins. GD2 expression was significantly higher in TNBC tumor tissues than in their matched normal tissues. Thus, GD2 could serve as a TNBC tumor marker (Ly et al., 2021). GD2 promotes cancer cell adhesion, proliferation, invasion, and metastasis by blocking the FAK signaling pathway (Shao et al., 2022). It also enhances TNBC tumor cell proliferation and tumor growth and metastasis by constitutively activating c-Met (Cazet et al., 2012). Dinutuximab beta is the first anti-GD2 mAb to be granted FDA approval as a neuroblastoma treatment. GD2-targeting CAR-T prevented tumor cell dissemination and lung metastasis in an *in situ* TNBC xenograft model. Third-generation GD2-CAR-T cells manifested significant antitumor immune responses including increased persistence and target cell lysis (Seitz et al., 2019). Clinical trials are being conducted to evaluate the safety and efficacy of GD2-CAR-T therapy so that it may advance from basic research to clinical application. Cancer Research UK conducted an anti-GD2-CAR-T Phase I cancer research trial (NCT02761915) on patients with relapsed or refractory neuroblastoma. The results indicated that targeting neuroblastoma with GD2-CAR-T cells might be a safe and efficacious therapeutic approach (Straathof et al., 2020). Del Bufalo et al. (2023) showed that the suicide gene in third-generation autologous GD2-CAR-T cells expressing inducible caspase 9 (GD2-CART01) is feasible and safe, and may have sustained efficacy against high-risk neuroblastoma (Del Bufalo et al., 2023).

### 3.7 Trophoblast cell -surface antigen 2 (TROP2)

TROP2, also known as tumor-associated calcium signal transducer (TACSTD2), was initially recognized as a trophoblast cell surface marker. TROP2 overexpression has been detected in 90% of all TNBCs (Dees et al., 2020). TROP2 influences tumor cell proliferation, migration, invasion, and metastasis by modulating the downstream signaling pathways MAPK (ERK1/2), JAK2/STAT3, and PI3K (Liu X. et al., 2022). Human antibodies targeting TROP2 inhibited tumor cell proliferation and migration, induced apoptosis, and suppressed breast cancer xenograft growth in a dose-dependent manner (Lin et al., 2014). TROP2 targeting is clinically safe and demonstrated very low toxicity in normal tissues. Therefore, it is promising as a therapeutic target in TNBC (Goldenberg et al., 2018). Recently, the FDA approved the antibody-drug sacituzumab govitecan that targets TROP2 and binds the topoisomerase-1 inhibitor SN-38. Sacituzumab govitecan is indicated as therapy for relapsed or metastatic TNBC in patients who have received at least two prior treatments (Seligson et al., 2021). CAR-T cells targeting TROP2 have been constructed for use in TNBC therapy. CD27 intracellular structural domain-based TROP2-CAR-T cells promoted relatively higher antitumor activity and T cell persistence in a mouse tumor-bearing model (Chen H. et al., 2021). As TROP2 targeting inhibits tumor growth, it could be clinically administered as TROP2-CAR-T cell therapy in the near future.

### 3.8 Epithelial cell adhesion molecule (EpCAM)

The transmembrane glycoprotein EpCAM is implicated in intercellular adhesion. EpCAM was upregulated in both primary and metastatic breast cancers, and silencing the EpCAM gene inhibited the proliferation, migration, and invasion of certain BC cell lines (Osta et al., 2004). EpCAM was closely associated with various vital signaling pathways as Wnt, transforming growth factor beta (TGF-β)/SMAD, EpEX/EGFR, and PI3K/Akt/mammalian target of rapamycin (mTOR) which regulate cancer cell adhesion, proliferation, invasion, and survival (Liu Y. et al., 2022). Granzyme B fusion proteins targeting EpCAM significantly inhibited tumor growth by ≤ 50% in mice inoculated with human TNBC cells (Amoury et al., 2016). Third-generation EpCAM-CAR-T cells were cytotoxic to target cells and induced lysis in them in an EPCAM-dependent manner leading to notable regression of tumor growth and secretion of cytotoxic interferon gamma (IFN-γ) and tumor necrosis factor alpha (TNF-α) in the MDA-MB-231 model. Zhang et al. (2019) validated the safety of EpCAM-targeting CAR-T cells (Zhang et al., 2019). A Phase I clinical trial (NCT02915445) is in progress to determine the safety of EpCAM-CAR-T cells in recurrent or refractory BC treatment. Blocking the tumor cell surface antigen EpCAM is a novel strategy for inhibiting tumor cell growth and metastasis and improving treatment efficacy in TNBC.

### 3.9 Stage-specific embryonic antigen-4 (SSEA-4)

SSEA-4 is a sphingolipid in human embryonic stem cells (hESCs). It is expressed in a tissue-specific manner and participates in cell proliferation, migration, senescence, apoptosis, and endocytosis (Sigal et al., 2022). Strong SSEA-4 upregulation in TNBC tumor cells indicates poor prognosis and chemotherapy resistance. Second-generation SSEA-4-CAR-T cells induced T cell degranulation, secreted cytokines, killed TNBC tumor cells, and demonstrated overall antitumor efficacy. An MDA-MB-231 xenograft mouse model displayed a significant reduction in TNBC tumor load despite a low antigen expression level (Rita and Pfeifer, 2018). Pfeifer et al. (2016) isolated healthy T cells using magnetic cells, activated SSEA-4-CAR-T with a colloidal nanosubstrate-based activation reagent (TransAct™), induced a tumor cell-killing effect (Pfeifer et al., 2016). Prior research showed that multiple rounds of CAR-T cell therapy enriched T cells in residual tumor cells. Hence, chemotherapy combined with CAR-T cell therapy should improve prognosis, survival, and outcome in patients with TNBC (Pfeifer et al., 2016).

## 4 Potential early clinical CAR-T targets in TNBC

### 4.1 Epidermal growth factor receptor (EGFR/HER1)

EGFR or HER1 is a receptor tyrosine kinase that activates oncogenic signaling pathways including rat sarcoma virus (Ras)/MAPK, PI3K/Akt, and phospholipase C (PLC)/PKC, and drives cancer cell growth, survival, and invasion (Sigismund et al., 2018). EGFR is overexpressed in ∼45–70% of all patients with TNBC (Hoadley et al., 2007). Third-generation CAR-T cells with an anti-EGFR antibody scFv region inhibited TNBC tumor growth by increasing cytokine secretion and cytolytic activity in mouse allograft models. The latter were established using Hs578T, MDA-MB-468, and MDA-MB-231 TNBC cell lines (Liu et al., 2019). A human glioblastoma study involved single dose of intravenously transfused autologous T cells that redirected CAR to EGFRvIII mutations. CART-EGFRvIII infusion presented significantly stronger antitumor and infiltrative effects than their untreated counterparts. Moreover, the treatment activated antigen loss and immunosuppressive counterattack mechanisms in the tumors. The approach was empirically feasible and safe and did not trigger exotoxicity or cytokine release syndrome (CRS) (O'Rourke et al., 2017). Clinical trials on targeted EGFR-CAR-T cell immunotherapy against TNBC are currently underway, and this treatment approach is expected to be routinely administered presently.

### 4.2 C-Met

C-Met, also known as hepatocyte growth factor receptor (HGFR), is a receptor tyrosine kinase that activates the RAS/MAPK and PI3K/Akt signaling pathways and mediates tumor cell proliferation, angiogenesis, migration, and metastasis (Breen et al., 2020). C-Met overexpression indicates poor prognosis in patients with TNBC. Silencing c-Met with small-molecule interfering RNAs (siRNAs) substantially reduced TNBC cell proliferation and migration (Kim et al., 2014). CAR-T constructs introduced by mRNA mitigated the off-tumor effect of targeting c-Met and connective tissue hyperplasia toxicity. Furthermore, intratumoral mRNA-c-Met-CAR-T cell injection was well tolerated and persistent, and induced an inflammatory response within the tumors (Tchou et al., 2017). Bifunctional CAR-T cells targeting c-Met and blocking programmed cell death protein 1 (PD-1) and programmed cell death protein ligand 1 (PD-L1) binding exerted stronger solid tumor antitumor activity and T cell persistence than single-target CAR-T cells in a tumor cell xenograft model (Yuan et al., 2021). Intratumoral injections of CAR-T cells targeting c-Met in advanced TNBC have passed the clinical trial stage and have demonstrated good safety and satisfactory tolerability (Toulouie et al., 2021). Therefore, targeting c-Met provides a potential target for the treatment of TNBC.

### 4.3 Mesothelin (MSLN)

MSLN is a glycoprotein on the surfaces of healthy mesothelial cells. It promotes tumor cell proliferation and migration and is anti-apoptotic as it induces the nuclear factor kappa-light-chain enhancer of activated B cells (NF-κB), MAPK, and PI3K signaling pathways (Morello et al., 2016). MSLN was overexpressed in ∼67% of all TNBC tissue specimens (Yang et al., 2021). As MSLN has high immunogenicity and low expression levels in normal tissues, targeting it is a potential therapeutic approach for TNBC. Li et al. (2020) successfully used CAR-T cells to target MSLN. This mechanism promoted tumor cell regression by inducing cytotoxicity and cytokine production. Hence, third-generation MSLN-CAR-T cells displayed long-term antitumor efficacy against MDA-MB-231 BC cells *in vitro*. MSLN-CAR-T cell therapy also inhibited MDA-MB-231 BC xenograft growth and liver metastasis *in vivo* (Li et al., 2020). Fourth-generation anti-MSLN-CAR-T cells induced significant T cell infiltration which, in turn, caused the regression of pre-established solid tumors and extended survival in a mouse tumor xenograft model (Adachi et al., 2018). Clinical trials on targeted MSLN-CAR-T cell immunotherapy against TNBC are currently underway, and this treatment approach is expected to be routinely administered presently.

### 4.4 Mucin 1 glycoprotein (MUC1)

MUC1 is a transmembrane glycoprotein localized to the upper surfaces of epithelial cells. It creates a protective mucosal barrier that prevents host cell infection, activates intracellular signaling pathways such as Ras/MAPK, JAK/STAT, and PI3K/Akt/mTOR, and promotes cancer cell adhesion, proliferation, differentiation, migration, and angiogenesis (Chen W. et al., 2021). MUC1-type *O*-linked *N*-acetylgalactosamine (GalNAc) is one of the most abundant forms of protein glycosylation in eukaryotes. The glycan moieties therein are attached via numerous Ser- and Thr-enriched tandem repeat sequence (VNTR) regions that serve as *O*-polysaccharide attachment scaffolds. This structure produces representative tumor antigens and makes MUC1 a good immunotherapy target (Sewell et al., 2006; Nath and Mukherjee, 2014). All BC cell subtypes and 95% of all TNBCs overexpress an aberrant glycosylated tumor form of MUC1 (tMUC1) that is not significantly expressed in normal breast cells and tissues. Second-generation MUC1-CAR-T cells exhibited relatively high tumor antigen specificity, strong tumor cytolytic activity, and elevated cytokine production. It also inhibited TNBC cell growth (Zhou et al., 2019). Tn-MUC1 is another abnormally glycosylated form of MUC1 that is abundantly expressed in BC (Zhou et al., 2018). Clinical trials are currently in progress to establish the safety and tolerability of MUC1-CAR-T cell therapy.

### 4.5 Receptor-tyrosine-kinase-like orphan receptor 1 (ROR1)

ROR1 is a protein that accelerates neuronal cell growth, activates intracellular signaling pathways such as ERK1/2, NF-κB, and nuclear factor erythroid 2-related factor 2 (NRF2), and enhances cancer cell migration, proliferation, and invasion (Kipps, 2022). ROR1 upregulation was detected in several malignancies including TNBC. In contrast, ROR1 expression levels are relatively low in healthy cells and tissues. ROR1-directed immunotherapy was nontoxic to vital normal organs in nonhuman primates. Wallstabe et al. (2019) found that ROR1-CAR-T cells actively entered arterial media flow, adhered to the tumor mass, infiltrated it, and elicited an antitumor response in advanced microphysiological 3D tumor models (Wallstabe et al., 2019). Compared to ROR1-CARs cells without short “Hinge-only” extracellular spacer, those with the foregoing structure more effectively lysed ROR1 (+) tumor cells and potentiated T cell effector function against primary chronic lymphocytic leukemia (CLL) and ROR1 (+) epithelial cancer lines without triggering activation-induced T cell death (Hudecek et al., 2013). ROR1-specific antibodies or efflux pump inhibitors can reverse chemotherapy resistance and tumor recurrence caused by ROR1-induced ATP-dependent drug efflux pump (ABCB1) expression in BCs (Fultang et al., 2020). The administration of ROR1-CAR-T cell therapy against TNBC has already been subjected to clinical efficacy validation trials.

### 4.6 Natural killer group 2, member D (NKG2D)

NKG2D is a transmembrane glycoprotein on NK cell surfaces. It detects stress ligands on infected or transformed cells and removes them via a cytotoxic mechanism. Thus, NKG2D regulates cytotoxicity, cytokine production, and survival (Jin F. et al., 2019). The NKG2D receptor/NKG2D ligand (NKG2DL) affects immune monitoring. Whereas the NKG2D receptor is activated by ligands expressed on stressed and transformed cells, NKG2DL is not detected in normal cells or tissues. NKG2D co-stimulates T cell receptor (TCR)-mediated effector molecule functions and potentiates antigen-specific, T cell-mediated cytotoxicity (Zloza et al., 2012). NKG2DL mediates actin reorganization, degranulation, and cytotoxicity as well as the activation of signaling pathways implicated in cytokine release including PI3K, NF-κB, and MEK/ERK (Chitadze and Kabelitz, 2022). NKG2DL-induced NKG2D associated with DNAX-activating protein 10 (DAP10) promotes actin recombination, degranulation, and cytotoxicity and the PI3K, NF-κB, and MEK/ERK signaling pathways (Chitadze and Kabelitz, 2022). The NKG2D ligand is only expressed in BC and especially on TNBC cells (Dees et al., 2020). For this reason, it is a potential TNBC immunotherapy target. Second-generation NGK2D-CAR-T cells with a 4-1BB or CD27 co-stimulatory structural domain increased T cell persistence and tumor regression and demonstrated strong antitumor activity in TNBC cell cultures and mouse xenogeneic models (Han et al., 2018). Cheng Wei et al. (2022) constructed NKG2D CAR-T cells that activated both 4-1BB and DAP10 co-stimulatory signals and realized low differentiation and depletion as well as the abundant proliferation of CAR-T cells both *in vitro* and *in vivo* (Wei et al., 2022). As NKG2D is a novel potential tumor antigen target, it is being evaluated in clinical trials. The results of experiments and trials performed to date suggest that NKG2D-CAR-T cells could improve the clinical prognosis of TNBC.

## 5 Clinical trial of CAR-T cell therapy in TNBC

Several clinical trials on TNBC therapy have targeted various antitumor antigens. Table 1 lists reports curated from https://clinicaltrials.gov/that pertained to the administration of CAR-T cells against TNBC. Most clinical trials cited in these studies are either in Phase I or Phase II. In 2013, the first TNBC CAR-T clinical trial (NCT01837602), which evaluated the safety of c-Met-CAR-T cells for the treatment of metastatic breast cancer and/or TNBC, was completed and the results showed that intratumoral injections of mRNA c-Met-CAR T cells was well tolerated by the tumor and elicited an inflammatory response in TNBC. The study showed that intratumoral c-Met-CAR-T cell infusion was well tolerated and elicited a pro-inflammatory response in TNBC tumors (Tchou et al., 2017). The People’s Liberation Army General Hospital began recruiting individuals for a Phase I trial determining the safe dose of meso-CAR-T cell therapy for patients with relapsed and/or chemorefractory TNBC (NCT02580747). The safety and efficacy of autologous MUC1-CAR-T cells in patients with advanced refractory TNBC are being validated in a Phase I/II trial (NCT02587689). The current status of the preceding trials is unknown. A Phase I dose-escalation trial (NCT02706392) was conducted on patients with TNBC to validate the safety of ROR1-CAR-T cell therapy. Combination oxaliplatin (Ox)/cyclophosphamide (Cy)/anti-PD-L1 therapy synergistically improved the infiltration and ameliorated the dysfunction of ROR1-CAR-T cells in TNBC tumors. Therefore, the foregoing protocol could augment the efficacy of CAR-T cell therapy (Srivastava et al., 2021). Tmunity Therapeutics initiated a phase 1 study of TnMUC1-targeted genetically-modified chimeric antigen receptor T cells in patients with advanced TnMUC1-positive solid tumors and multiple myeloma (NCT04025216). This study method is that patients receive CART-TnMUC1 treatment after receiving cyclophosphamide and fludarabine lymphodepletion chemotherapy, which is similar to the T4 immunotherapy phase 1 trial method for Head and neck cancer initiated by King’s College London (NCT01818323). A study (NCT04025216) initiated by Tmunity Therapeutics was terminated as TnMUC1-CAR-T cell therapy presented certain safety, tolerability, and feasibility risks in patients with TNBC patients. An open-label, single-center, Phase I dose-escalation study (NCT04107142) was conducted to validate the safety and tolerability of chimeric antigen receptor-transplanted gamma delta (γδ) T cells (CTM-N2D) targeting haploidentical or allogeneic NKG2DL in subjects with relapsed or refractory solid tumors. The Second Affiliated Hospital of Guangzhou Medical University has enrolled patients with advanced TNBC in a Phase I trial (NCT05341492) to validate the safety and efficacy of EGFR/B7-H3 CAR-T. Lyell Immunopharma lnc. has recruited TNBC patients with ROR1+ relapsed or refractory TNBC to validate the safety and tolerability of LYL797 (NCT05274451), which refers to ROR1-targeted CAR-T cells. The increasing number of TNBC CAR-T therapies in clinical trials will set the stage for a new era of TNBC immunotherapy.

**TABLE 1:** Ongoing clinical trials of CAR-T cell therapy for triple-negative breast cancer.

CAR-T cell target	Clinical trial number	State	Phase	Sponsor	Clinical trial of CAR-T cell therapy in TNBC
c-Met	NCT01837602	Completed	Phase 1	University of Pennsylvania	Clinical Trial of Autologous cMet Redirected T Cells Administered Intratumorally in Patients With Breast Cancer
MSLN	NCT02580747	Unknown	Phase 1	Chinese PLA General Hospital	Clinical Study of Chimeric Mesothelin Antigen Receptor-modified T Cells in Relapsed and/or Chemotherapy Refractory Malignancies
MUC1	NCT02587689	Unknown	Phase 1/2	PersonGen BioTherapeutics (Suzhou) Co., Ltd.	Phase I/II Study of Anti-MUC1 CAR T Cells for Patients With MUC1+ Advanced Refractory Solid Tumor
ROR1	NCT02706392	Terminated	Phase 1	Fred Hutchinson Cancer Center	Phase I Study of Adoptive Immunotherapy for Advanced ROR1+ Malignancies With Defined Subsets of Autologous T Cells Engineered to Express a ROR1-Specific Chimeric Antigen Receptor
TnMUC1	NCT04025216	Terminated	Phase 1	Tmunity Therapeutics	A Phase 1 Open-Label, Multi-Center First in Human Study of TnMUC1-Targeted Genetically-Modified Chimeric Antigen Receptor T Cells in Patients With Advanced TnMUC1-Positive Solid Tumors and Multiple Myeloma
NKG2DL	NCT04107142	Unknown	Phase 1	CytoMed Therapeutics Pte Ltd	A Phase I Dose-escalation Trial to Evaluate Haploidentical/Allogeneic Natural Killer Group 2D Ligand (NKG2DL)-Targeting Chimeric Antigen Receptor-grafted Gamma Delta (γδ) T Cells (CTM-N2D) in Subjects With Relapsed or Refractory Solid Tumour
EGFR/B7H3	NCT05341492	Recruiting	Phase 1	Second Affiliated Hospital of Guangzhou Medical University	A Single-arm, Open, Exploratory Clinical Study Evaluating the Safety and Efficacy of EGFR/B7H3 CAR-T in Patients With EGFR/B7H3-positive Advanced Solid Tumors (Lung and Triple-negative Breast Cancer)
ROR1	NCT05274451	Recruiting	Phase 1	Lyell Immunopharma, Inc.	A Phase 1 Study to Assess the Safety and Efficacy of LYL797, ROR1-Targeting CAR T Cells, in Adults With Relapsed and/or Refractory Solid-Tumor Malignancies

## 6 Solid tumor dilemma and new remedial strategies involving CAR-T

Of the eight CAR-T products approved worldwide, six target CD19, two target B cell maturation antigen (BCMA), and all effectively treat hematological malignancies (Table 2). The administration of CAR-T for hematological tumor therapy has rapidly progressed. In contrast, the application of CAR-T in the management of solid tumors has advanced far more slowly. Nevertheless, solid tumors account for >90% of all cancers (Siegel et al., 2020). Therefore, the practical clinical application of CAR-T in solid tumor treatment is severely limited.

**TABLE 2:** Overview of 8 marketed CAR-T products for the treatment of different hematologic malignancies.

Drug name	Target	Enterprise	Time of approval	Generation (intracellular signal domain)	Indications (approved treatment line)	Relevant regulatory agency
Kymriah	CD19	Novartis	August 2017	2^nd^(4-1BB-CD3ζ)	B-ALL (3^rd^ line) DLBCL (3^rd^line)	FDA, EMA and PMDA
					FL (3^rd^line)	
Yescarta	CD19	Kite/Gilead	October 2017	2^nd^(CD28-CD3ζ)	DLBCL (2^nd^line)	FDA, EMA and PMDA
					FL (3^rd^line)	
Tecartus	CD19	Kite/Gilead	July 2020	2^nd^(CD28-CD3ζ)	MCL (3^rd^line)	FDA and EMA
					B-ALL (3^rd^line)	
Breyanzi	CD19	BMS/Juno	February 2021	2^nd^(4-1BB-CD3ζ)	DLBCL (3^rd^line)	FDA, EMA and PMDA
Ekeda (Aquilenside Injection)	CD19	Fosun Kate	June 2021	2^nd^(CD28-CD3ζ)	DLBCL (3^rd^line)	NMPA
Benodar (Regiorense Injection)	CD19	Yao Ming Ju Nuo	September 2021	2^nd^(4-1BB-CD3ζ)	MCL (3^rd^ line) DLBCL (3^rd^line)	NMPA
Abecma	BCMA	BMS/Blue bird	March 2021	2^nd^(4-1BB-CD3ζ)	MM (5^th^line)	FDA, EMA and PMDA
Carvykti (Siddaggi Orense)	BCMA	Legend/JNJ	February 2022	2^nd^(4-1BB-CD3ζ)	MM (5^th^line)	FDA and EMA

FDA, food and drug administration; EMA, european medicines agency; NMPA, state medical products administration; PMDA, japan pharmaceutical and medical devices agency.

### 6.1 Tumor heterogeneity

Many malignant solid tumors are highly heterogeneous. The extensive division and proliferation that occur during tumor growth alter the molecular biology and genetic profiles of the subsequent generations and cause wide variability in tumor growth rate, invasion ability, drug sensitivity, and disease prognosis. While most tumor-associated antigens (TAAs) such as CD9, CD29, CD49c, and integrin β5 are upregulated on BC cells, they nonetheless occur on normal breast cells as well (Remsik et al., 2018; Qiu et al., 2020). In the presence of tumor-specific antigens (TSAs), TAAs contribute to tumor heterogeneity, and selective CAR-T targets allow antigen-negative tumor cells to escape (Guedan et al., 2019). In recent years, researchers investigated whether two or more different tumor antigens may simultaneously prevent TNBC relapse resulting from CAR-T cell therapy failure caused by antigen downregulation or loss. After fully mature T cells are activated, they can only exert a tumor-killing effect. A few preclinical study on models demonstrated that the simultaneous administration of several CAR constructs could target multiple tumor antigens conferring antigen recognition multispecificity (Hegde et al., 2013; Bielamowicz et al., 2018). The most extensively studied dual CARs included single-target CAR mixes and bivalent tandem, bivalent cyclic, and dual *cis-trans* CARs. Other studies explored the development of novel CAR structures. One study revealed that bicistronic CD19/CD22 CAR-T cells exhibited relatively low persistence whereas dual CARs targeting different co-stimulatory domains tailored to each antigen were comparatively more effective at decreasing toxicity and increasing antigen sensitivity (Kokalaki et al., 2023). Ongoing clinical trials are evaluating dual (Clinical Trials. gov: NCT03330691) and tandem (Clinical Trials. gov: NCT03185494, NCT03097770, and NCT03019055) CAR-T cells targeting either CD19 and CD22 or CD20 for leukemia and lymphoma. This approach will eventually be applied to solid tumors such as BCs.

### 6.2 Tumor microenvironment (TME) immunosuppression

The current consensus is that the TME comprises tumor, immune, and endothelial cells as well as fibroblasts, secreted cytokines, and ECM proteins. Intricate interactions among the specialized elements in the TME contribute to the resilience and low immunogenicity of tumors and create the characteristic internal conditions, namely, hypoxia, low Trp, Lys, and Arg content, and high levels of organic acids generated by anaerobic metabolism (Huang et al., 2018). Under hypoxic conditions, cancer cells and tumor-associated macrophages (TAMs) inhibit T lymphocyte proliferation by releasing high concentrations of prostaglandin E2 (PGE2), adenosine-activated, G protein-coupled receptors, and protein kinase A (D'Aloia et al., 2018). Regulatory T cells (Tregs), MDSCs, TAMs, and tumor-associated neutrophils (TANs), transforming growth factor-β (TGF-β), IL-10, and indoleamine 2,3-dioxygenase (IDO), which attenuate immune effector cell activity, thereby improving immunosuppression and tolerance (Li et al., 2018; Hanna and Balko, 2021). T cells expressing dominant negative TGF-β receptors were constructed to activate CAR-T cells in this context (Foster et al., 2008). In another strategy, the immunosuppressive signal is converted into an immunostimulatory signal via chimeric cytokine receptors that bind the outer domain of the receptor of an immunosuppressive factor such as IL-4 to the intracellular domain of the IL-2/IL-7 receptor. In this manner, T cell persistence and antitumor activity within the tumor are increased (Wilkie et al., 2010). Various combinations of CAR-T cells and checkpoint inhibitors such as anti-PD1 and anti-PD-L1 blockers or other therapeutic agents targeting the immunosuppressive microenvironment have greater therapeutic efficacy than monotherapy, can improve outcomes and prognoses, and overcome the obstacles associated with solid TMEs (John et al., 2013). CAR-T cells combined with cross-linked multilayer liposome vesicles (cMLV) bearing the A2aR-specific small molecule antagonist SCH-58261 penetrated immunosuppressive TMEs and corrected intratumoral T cell hypofunction (Siriwon et al., 2018).

### 6.3 CAR-T transport and infiltration

Unlike scattered blood cancer cells, solid tumors such as BCs form solid masses that collaborate with CAFs and blood vessels to create physical T cell traffic barriers. Certain solid tumors also inhibit chemokine secretion and prevent binding between the chemokine receptors on the surfaces of CAR-T cells and the chemokines present in tumor cells or TME (Zhong et al., 2023). As the surfaces of CAR-T cells lack receptors matching the chemokines secreted by solid tumors, CAR-T cells cannot accurately navigate to target tumor sites (Harlin et al., 2009). However, intratumoral administration improves CAR-T infiltration and transport, eliminates the requirement for CAR-T cell transit to the tumor lesion, and mitigates the toxicity of targeted connective tissue hyperplasia. Intratumoral administration has been evaluated for malignant glioma and pleural mesothelioma and is being validated in clinical trials (NCT02208362, NCT02414269) (Adusumilli et al., 2014; Brown et al., 2018). Once unique chemokines are identified, they may be deployed to enable T cells to recognize specific tumor targets. In this way, the accuracy with which CAR-T cells attack solid tumors may be improved. Integrin αvβ6 targeting, IL-8 receptor overexpression, and C-X-C motif chemokine receptor 1 (CXCR1) or CXCR2 modification can enhance the traffic and increase the cytotoxicity of CAR-T cells (Jin L. et al., 2019; Phanthaphol et al., 2021). CARs constructed with acetyl heparinase to degrade the tumor cell ECM and those that target fibroblast-activating protein (FAP) showed superior tumor infiltration and antitumor activity compared to their unmodified CAR counterparts (Wang et al., 2014; Caruana et al., 2015). Anti-angiogenic agents can facilitate CAR-T cell transit to target tumor sites possibly by targeting the repair of “spontaneous” DNA double-strand breaks caused by recombination-activating gene 1/2 (RAG1/2) and/or reactive oxygen species (ROS), thereby augmenting the therapeutic efficacy of standard cytotoxic drugs (Dasgupta et al., 2018).

### 6.4 Endogenous T cell suppressor signaling

Endogenous T cell suppressor signaling weakens the antitumor capacity of CAR-T. After CAR-T cells undergo antigen activation, PD-1 and cytotoxic T lymphocyte-associated protein 4 (CTLA-4) suppress T cell proliferation and cytokine secretion by binding the required ligands (Parry et al., 2005). Gene silencing as well as PD-1 inhibitors and switch receptors are used mainly to block endogenous inhibitory signals. Positive CAR stimulation may be combined with the blockade of the negative T cell regulators PD-1 and CTLA-4 mediated by Cas9 ribonucleoprotein (RNP) destruction. Together, they potentiate the control of TBNC by T cells (Hu et al., 2019). The mutation of PD-1 immune receptor tyrosine-based switch motif (ITSM) significantly inhibited the growth of xenograft tumors by enhancing the antitumor activity of cytotoxic T cells, and did not affect the secretion of cytokines by T cells, nor did it induce T cell apoptosis (Qi et al., 2020). Immune checkpoint inhibitors (ICIs) are highly effective as they target inhibitory co-stimulatory molecules such as PD-1 and CTLA-4 on CAR-T lymphocyte surfaces, improve tumor control, and maintain T cell persistence (Sharma and Allison, 2015).

### 6.5 CAR-T-related toxicity

The toxicity of CAR-T cells partially determines their therapeutic efficacy. When CAR-T cells destroy tumor cells, the tumor components are released into the adjacent and surrounding tissues, immune cells are rapidly activated, systemic inflammatory cytokines such as IL-6, IFN-γ, and C-reactive protein (CRP) are secreted, and cytokine release syndrome (CRS), neurotoxicity and in severe cases, death may ensue (Chou and Turtle, 2020). Differential TAA expression among various tumor types makes it difficult to identify optimal target surface antigens. Cross-reactivity between CAR-T cells and normal tissue during tumor targeting and detumorization can lead to lethal toxicity. Various approaches that simultaneously validate CAR-T therapy efficacy and safety are being investigated. Tocilizumab is a monoclonal anti-human IL-6 receptor antibody that was originally approved to treat rheumatic diseases. It was discovered that it also lowers the risk of CRS (Kotch et al., 2019). Tocilizumab and certain other mAbs have been confirmed for use in CRS salvage therapy. When inhibitory CARs (iCARs) are combined with specific antigens expressed only in normal tissues, they trigger inhibitory signals and increase the efficacy and decrease the toxicity of solid tumor therapy (Fedorov et al., 2013). Another strategy involves the administration of protease-based, small molecule-assisted shutdown CARs (SWIFF-CARs) (Juillerat et al., 2019). Binding herpes simplex virus thymidine kinase (HSV-TK) and inducible cysteine aspartase 9 (iCasp9) to suicidal genes significantly attenuated CAR-T cell cytotoxicity (Zhou et al., 2015). The introduction of suicide genes into CAR-T cells via a “transient CAR-T cell” method also improves the safety of CAR-T cell therapy (Yu et al., 2019).

### 6.6 CAR-T in combination with other therapies to treat TNBC

CAR-T therapy alone has limited efficacy against complex TNBC. Hence, the antitumor effect, expansion, and persistence of CAR-T must be fortified. CAR-T in combination with traditional radiotherapy, chemotherapy, ICIs, lysing viruses, Bruton tyrosine kinase (BKT) inhibitors, and other technologies may help guide T cells toward their cancer cell targets, thereby improving therapeutic efficacy. Hence, the preceding modalities have good value in clinical applications. The use of CAR-T therapies in combination with other treatment modalities simultaneously targeting different mechanisms improved clinical outcomes in solid tumors treated with CAR-T (Yang et al., 2022). Though CAR-T cell therapy is unlikely to replace chemotherapy for TNBC treatment anytime soon, it may nonetheless be used in combination therapy. The FDA recently approved the combination of atezolizumab and albumin-bound paclitaxel for the treatment of PD-L1-positive, locally advanced, or metastatic unresectable TNBC (Schmid et al., 2020). CAR based on the CD32-Fc receptor (CD32A 131R CAR) eliminates TNBC cells by modulating their affinity for mAbs such as cetuximab and panitumumab and secreting IFN-γ and TNF-α (Caratelli et al., 2020). Furthermore, the combination of CAR-T cell therapy plus immune checkpoints such as anti-PD-1 resists the TME and enhances cytotoxicity and persistence against intratumor T cells (Corti et al., 2022). Targeted Fra-1 inhibition sensitized TNBC cells to the poly (ADP-ribose) polymerase 1 (PARP1) inhibitor olaparib. Thus, FRa-1 targeting combined with olaparib administration might significantly improve outcomes in patients with TNBC (Song et al., 2021).

**FIGURE 3:**
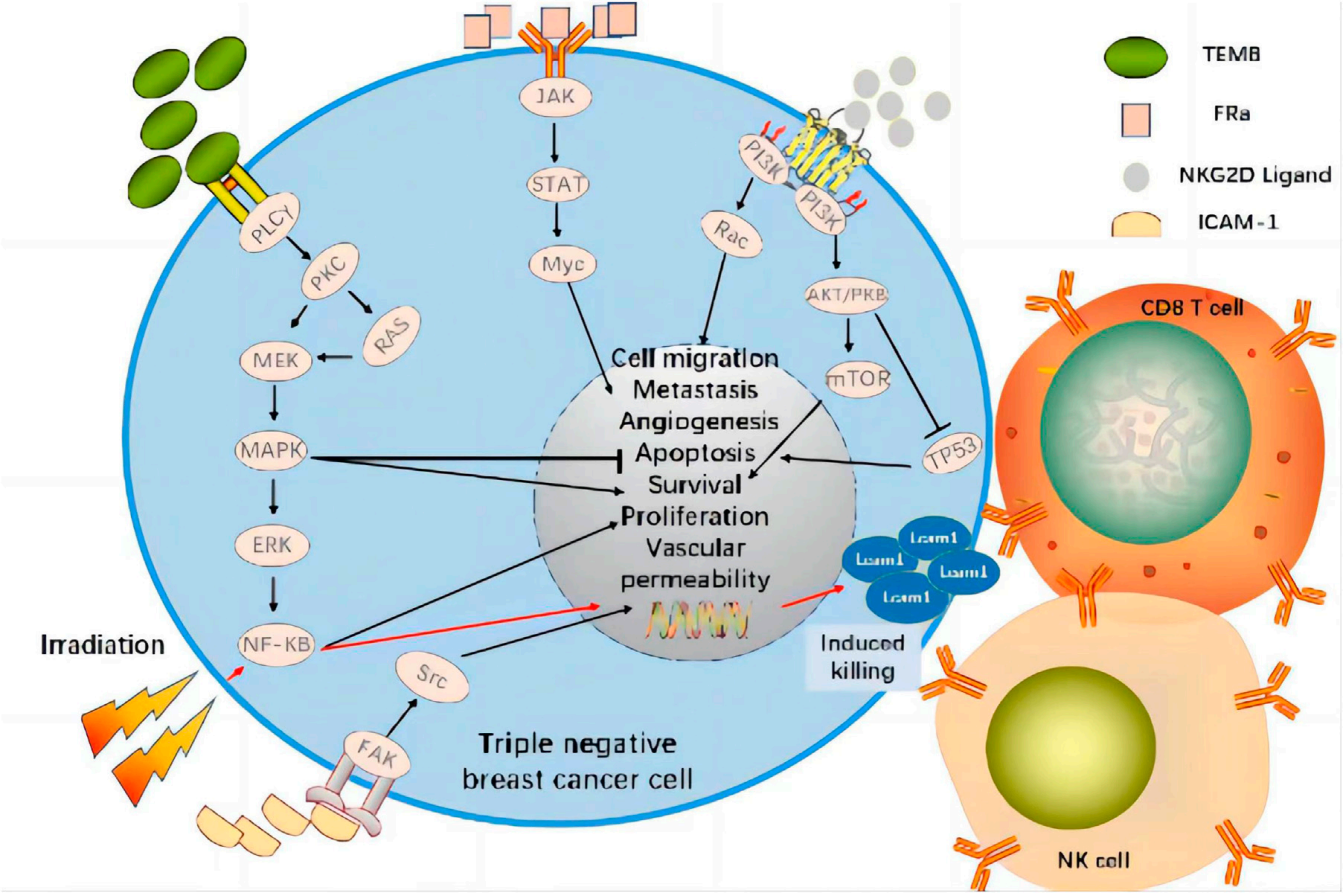
CAR Targets and Signaling Pathways Affected by Radiotherapy in Clinical Trial of CAR-T Cell Therapy for TNBC. The combination of targeted tumor antigen CAR-T cell therapy and radiotherapy significantly upregulates ICAM-1 on TNBC cells by activating NF-kB signaling and promoting CD8+T and NK cell infiltration within the tumor. Therefore, it shows strong anti-tumor efficacy.

A recent study demonstrated that the combination of targeted EGFR CAR-T cell therapy and radiotherapy significantly upregulated ICAM-1 on TNBC cells by activating NF-kB signaling and, by extension, promoting intratumor CD8^+^ T and NK cell infiltration. Compared to CAR-T combined with fractionated radiotherapy, CAR-T in combination with single high-dose radiotherapy prolonged survival and presented stronger antitumor efficacy in immunologically active and immunodeficient TNBC mice *in situ* (Figure 3) (Zhou et al., 2022). In relapsed/refractory (R/R) B cell non-Hodgkin’s lymphoma (bNHL), high bridging radiotherapy (BRT) doses delivered within a narrow time window followed by CAR-T therapy improved local control rates. Remedial radiotherapy (SRT) administered after CAR-T therapy may also correct limited relapses in diseases treated with CAR-T (Saifi et al., 2022). This approach was initially successful for bNHL treatment and could potentially treat TNBC effectively as well. In a breast cancer gene (BRCA)-deficient TNBC model, olaparib activated the intratumoral cyclic guanosine monophosphate/adenosine monophosphate synthase (cGAS)/stimulator of interferon genes (STING) pathway, stimulated paracrine secretion by dendritic cells, and promoted CD8^+^ T cell infiltration, activation, and recruitment (Pantelidou et al., 2019). An *in vitro* study modulation of PI3K in CAR-T cells showed that PI3K induces a memory phenotype that improves tumor killing and T cell persistence in leukemia (Zheng et al., 2018). A preclinical study found that tissue factor (TF)-targeting, CAR-engineered natural killer (TF-CAR-NK) cells attacked and killed TNBC cells, and antibody-like immunoconjugate-mediated, antibody-dependent cellular cytotoxicity (L-ICON ADCC) enhanced their therapeutic efficacy. Cooperation between innate-like tissue-resident γδ T cell compartments and the adaptive responses mounted by αβ T cells conferred protection against carcinogenesis (Wu et al., 2019; Hu, 2020).

## 7 Conclusion and future perspectives

The present review briefly examined the historical evolution of triple-negative breast cancer, CAR-T cell immunotherapy, and CAR structures and explored 15 antigens FRA, TEM8, CSGP4, ICAM-1, AXL, GD2, TROP2, EpCAM, SSEA-4, EGFR, HGFR/c-MET, MSLN, MUC1, ROR1, and NKG2D) that CAR-T cells target in TNBC (Table 3). Preclinical trials on tumor antigens indicate that they have substantial antitumor and cytokine release activity. Certain tumor antigens have already entered human clinical trials (Table 1). Considerable progress has been made in the refinement and optimization of CAR-T cell therapies. Nevertheless, they still face numerous practical challenges including tumor heterogeneity, the immunosuppressive environment, inadequate transport and infiltration, endogenous T cell suppressive signaling, and toxicity. Here, we evaluated the relative efficacy of CAR-T therapy against solid tumors in general and TNBC in particular and addressed several TNBC-related target antigens. Selecting appropriate target antigens for anti-TNBC CAR-T therapy and improving its specificity, safety, and durability will determine its success. We also investigated various combinations of CAR-T cell therapy with conventional TNBC treatments. As combination therapies improved T cell tumor infiltration and persistence and had potent tumor-killing efficacy, they might be feasible as therapeutic approaches against TNBC. CAR-T cell construction, production, preservation, transportation, and individualized administration will merit serious consideration after CAR-T therapy is routinely applied in clinical settings. The findings of the present review also suggest that research institutions and pharmaceutical manufacturers should collaborate in the development, fabrication, testing, and validation of CAR-T cell therapy for the treatment of TNBC in particular and solid tumors in general.

**TABLE 3:** CAR-T cell targets in triple-negative breast cancer.

CAR-T cell target	Target category	Mediating signal transduction pathway
FRa	Cell surface glycoprotein	JAK-STAT
TEM8	Cell surface glycoprotein	ERK/Bcl-2
CSPG4	Cell surface glycoprotein	FAK, ERK/2, PKCα, AKT
ICAM-1	Cell surface glycoprotein	FAK/Src
AXL	Receptor tyrosine kinase (RTK)	PI3K, MAPK, JAK/STAT
GD2	Sialic ganglioside	FAK
TROP2	Cell surface glycoprotein	MAPK, JAK2/STAT3, PI3K
EpCAM	Cell surface glycoprotein	Wnt, TGF-β/SMAD, EpEX/EGFR, PI3K/AKT/mTOR
SSEA-4	Cell surface glycoprotein	FAK/Src
EGFR/HER1	Receptor tyrosine kinase (RTK)	Ras/MAPK, PI3K/AKT, PLC/PKC
c-Met/HGFR	Receptor tyrosine kinase (RTK)	RAS/MAPK, PI3K/AKT
MSLN	Cell surface glycoprotein	NF-κB, MAPK, PI3K
MUC1	Cell surface glycoprotein	Ras/MAPK, JAK/STAT, PI3K/Akt/mTOR
ROR1	Receptor tyrosine kinase (RTK)	ERK1/2, NF-kB, NRF2
NKG2D Ligand	Stress ligand	PI3K, NF-kB, MEK/ERK
